# Association of Frailty, Age, Socioeconomic Status, and Type of Surgery With Perioperative Outcomes in Patients Undergoing Noncardiac Surgery

**DOI:** 10.1001/jamanetworkopen.2022.24625

**Published:** 2022-07-29

**Authors:** Kai Yi Wu, Pishoy Gouda, Xiaoming Wang, Michelle M. Graham

**Affiliations:** 1 Mazankowski Alberta Heart Institute, Division of Cardiology, University of Alberta, Edmonton, Alberta, Canada; 2 Research Facilitation, Alberta Health Services, Edmonton, Alberta, Canada

## Abstract

This cohort study examines the association of Hospital Frailty Risk Score classification, demographic characteristics, and type of surgery with risk of mortality among patients undergoing noncardiac surgery.

## Introduction

Frailty is associated with poor clinical outcomes and is a consequence of cumulative multisystem decline in function and depletion of homeostatic reserves.^1,2^ The Hospital Frailty Risk Score (HFRS) screens for frailty using *International Classification of Diseases, Ninth Revision *(*ICD-9*) and *International Statistical Classification of Diseases and Related Health Problems, Tenth Revision *(*ICD-10*) codes.^3^ The HFRS has been used to identify patients undergoing noncardiac surgery at high risk for prolonged hospital stay, in-hospital mortality, and 1-year mortality.^4^ We sought to further explore the association of HFRS classification across subgroups of age, socioeconomic status (SES), and surgery type.

## Methods

This cohort study evaluated patients undergoing elective noncardiac surgery between October 1, 2008, and September 30, 2019, in Alberta, Canada.^4^ Of 798 918 patients undergoing noncardiac surgery, 201 706 were excluded because of a lack of postal codes, income data, preceding surgery in the year before, and undergoing an inpatient minor procedure. Socioeconomic status was calculated using an individual’s postal code to estimate SES from census data.^5^ Surgical risk level was defined by British United Provident Association operative grade. The HFRS was calculated using 109 *ICD-9* and *ICD-10* codes associated with frailty in any position, using a 2-year look-back period.^3^ The primary outcome was in-hospital mortality. Secondary outcomes included 30-day and 1-year mortality. This study complies with the Declaration of Helsinki^6^ and was approved by the University of Alberta Health Research Ethics Board with a waiver of informed consent because of the minimal risk for participants and deidentified data use. This study followed the STROBE reporting guideline.

Continuous variables were described using mean (SD) and median (IQR). The difference of a continuous variable across HFRS groups was tested using the Kruskal-Wallis rank test. Categorical variables were tabulated using number (percentage), and 2-sided *P* values were from χ^2^ tests. *P* < .05 was considered to be significant. Odds ratios (95% CIs) for mortality outcomes were obtained using multivariable logistic regression adjusted with sex, age, individual components of the Charlson Comorbidity Index, revised cardiac risk index, and surgical risk. Stepwise variable selection procedure was adopted to select important variables (with a default enter and stay criterion of .05). Statistical analysis was performed between March 28, 2021, and June 1, 2022, using SAS software, version 7.1 (SAS Institute Inc) and R software (R Foundation).

## Results

The final cohort consisted of 597 212 patients (mean [SD] age, 52.7 [22.4] years; 337 595 [56.5%] female) (Table 1). A total of 5528 in-hospital deaths (0.13%) occurred, with an additional 758 (0.13%) within 30 days of discharge and 8439 (1.43%) within 1 year of discharge. Across all subgroups of age, SES, and surgical risk, higher HFRSs were associated with increased in-hospital mortality (overall high vs low risk score: aOR, 9.34; 95% CI, 8.49-10.28; *P* < .001), 30-day mortality (overall high vs low risk score: aOR, 4.44; 95% CI, 3.51-5.62; *P* < .001), and 1-year mortality (overall high vs low risk score: aOR3.62; 95% CI, 3.34-3.92; *P* < .001) (Table 2).

**Table 1.** Characteristics of the Patient Population Stratified by HFRS^a^

Characteristic	HFRS category			Overall (N = 597 212)	*P* value
	Low risk (<5) (n = 530 477)	Medium risk (5-15) (n = 51 182)	High risk (>15) (n = 15 553)		
Age, y					
Mean (SD)	50.6 (21.9)	68.4 (19.3)	75.0 (15.9)	52.7 (22.4)	<.001
Median (IQR)	54.0 (37.0-67.0)	72.0 (58.0-83.0)	79.0 (67.0-86.0)	56.0 (38.0-69.0)	
Sex					
Female	303 124 (57.1)	25 954 (50.7)	8517 (54.8)	337 595 (56.5)	<.001
Male	227 353 (42.9)	25 228 (49.3)	7036 (45.2)	259 617 (43.5)	
HFRS					
Mean (SD)	0.6 (1.1)	8.7 (2.8)	21.2 (5.7)	1.8 (4.2)	<.001
Median (IQR)	0.0 (0.0-0.5)	8.1 (6.3-10.8)	19.6 (17.0-23.8)	0.0 (0.0-1.5)	
CCI score					
Mean (SD)	0.3 (0.6)	1.0 (1.1)	1.5 (1.2)	0.4 (0.7)	<.001
Median (IQR)	0.0 (0.0-1.0)	1.0 (0.0-2.0)	1.0 (1.0-2.0)	0.0 (0.0-1.0)	
CCI score category					
0	396 390 (74.7)	20 205 (39.5)	3509 (22.6)	420 104 (70.3)	<.001
1	99 035 (18.7)	16 856 (32.9)	5491 (35.3)	121 382 (20.3)	
≥2	35 052 (6.6)	14 121 (27.6)	6553 (42.1)	55 726 (9.3)	
No. of admissions in preceding 2 y					
1	418 409 (78.9)	18 562 (36.3)	2681 (17.2)	439 652 (73.6)	<.001
2	84 189 (15.9)	15 281 (29.9)	3882 (25.0)	103 352 (17.3)	
≥3	27 879 (5.3)	17 339 (33.9)	8990 (57.8)	54 208 (9.1%)	
RCRI score					
0	487 376 (91.9)	29 187 (57.0)	5938 (38.2)	522 501 (87.5)	<.001
1	35 221 (6.6)	13 620 (26.6)	4908 (31.6)	53 749 (9.0)	
2	1159 (0.2)	1927 (3.8)	1347 (8.7)	4433 (0.7)	
≥3	6721 (1.3)	6448 (12.6)	3360 (21.6)	16 529 (2.8)	
Surgery risk					
Intermediate (BUPA 2)	141 017 (26.6)	15 229 (29.8)	5043 (32.4)	161 289 (27.0)	<.001
Major (BUPA 3)	204 739 (38.6)	21 870 (42.7)	7763 (49.9)	234 372 (39.2)	
Major plus (BUPA 4)	156 862 (29.6)	11 552 (22.6)	2222 (14.3)	170 636 (28.6)	
Complex major (BUPA 5)	27 859 (5.3)	2531 (4.9)	525 (3.4)	30 915 (5.2)	

Abbreviations: BUPA, British United Provident Association; CCI, Charlson Comorbidity Index; HFRS, Hospital Frailty Risk Score; RCRI, Revised Cardiac Risk Index.

^a^ Data are presented as number (percentage) of patients unless otherwise indicated.

**Table 2.** Association of HFRS, Sex, Age, Surgical Risk, and SES With Mortality

HFRS category	In-hospital mortality		30-d Mortality		1-y Mortality	
	aOR (95% CI)^a^	*P* value	aOR (95% CI)^a^	*P* value	aOR (95% CI)^a^	*P* value
Overall						
Medium risk vs low risk	7.55 (7.01-8.13)	<.001	3.69 (3.09-4.42)	<.001	2.72 (2.57-2.88)	<.001
High risk vs low risk	9.34 (8.49-10.28)	<.001	4.44 (3.51-5.62)	<.001	3.62 (3.34-3.92)	<.001
Sex						
Male						
Medium risk vs low risk	6.91 (6.27-7.61)	<.001	2.79 (2.21-3.52)	<.001	2.49 (2.31-2.69)	<.001
High risk vs low risk	8.9 (7.83-10.10)	<.001	3.57 (2.61-4.88)	<.001	3.31 (2.96-3.69)	<.001
Female						
Mediate risk vs low risk	8.27 (7.36-9.28)	<.001	5.5 (4.15-7.28)	<.001	3.01 (2.75-3.28)	<.001
High risk vs low risk	9.54 (8.25-11.03)	<.001	6.35 (4.44-9.07)	<.001	3.98 (3.54-4.47)	<.001
Age, y						
<65						
Medium risk vs low risk	16.45 (14.31-18.92)	<.001	8.26 (5.93-11.51)	<.001	4.64 (4.12-5.22)	<.001
High risk vs low risk	27.83 (22.91-33.81)	<.001	9.07 (4.88-16.86)	<.001	9.97 (8.33-11.94)	<.001
65-69						
Medium risk vs low risk	11.28 (8.98-14.16)	<.001	8.36 (5.25-13.32)	<.001	3.12 (2.62-3.72)	<.001
High risk vs low risk	12.82 (9.42-17.45)	<.001	10.49 (4.86-22.61)	<.001	4.9 (3.75-6.39)	<.001
70-74						
Medium risk vs low risk	7.22 (5.88-8.86)	<.001	6.27 (3.88-10.14)	<.001	2.77 (2.36-3.25)	<.001
High risk vs low risk	9.01 (6.81-11.92)	<.001	15.86 (8.85-28.42)	<.001	4.91 (3.9-6.17)	<.001
75-79						
Medium risk vs low risk	6.24 (5.16-7.54)	<.001	3.04 (1.86-4.97)	<.001	2.58 (2.24-2.98)	<.001
High risk vs low risk	8.66 (6.83-10.98)	<.001	5.42 (2.96-9.92)	<.001	3.05 (2.47-3.76)	<.001
80-84						
Medium risk vs low risk	4.48 (3.73-5.38)	<.001	3.64 (2.41-5.50)	<.001	2.16 (1.89-2.46)	<.001
High risk vs low risk	6.03 (4.86-7.48)	<.001	4.86 (2.96-7.96)	<.001	2.8 (2.36-3.32)	<.001
≥85						
Medium risk vs low risk	2.37 (2.05-2.74)	<.001	1.73 (1.24-2.40)	.001	1.39 (1.24-1.57)	<.001
High risk vs low risk	2.65 (2.23-3.15)	<.001	2.19 (1.50-3.18)	<.001	1.51 (1.3-1.74)	<.001
Surgery risk						
Intermediate						
Medium risk vs low risk	10.42 (8.94-12.16)	<.001	5.8 (4.22-7.97)	<.001	2.93 (2.64-3.25)	<.001
High risk vs low risk	14.68 (12.19-17.68)	<.001	7.84 (5.31-11.58)	<.001	3.76 (3.29-4.31)	<.001
Major						
Medium risk vs low risk	5.93 (5.19-6.77)	<.001	4.79 (3.45-6.65)	<.001	2.79 (2.51-3.09)	<.001
High risk vs low risk	6.31 (5.38-7.40)	<.001	5.03 (3.37-7.51)	<.001	3.47 (3.05-3.96)	<.001
Major plus						
Medium risk vs low risk	7.21 (6.42-8.10)	<.001	2.49 (1.78-3.48)	<.001	2.26 (2.03-2.52)	<.001
High risk vs low risk	10.04 (8.49-11.88)	<.001	3.13 (1.85-5.31)	<.001	3.27 (2.72-3.94)	<.001
Complex						
Medium risk vs low risk	10.19 (7.71-13.45)	<.001	2.7 (1.34-5.43)	.005	2.38 (1.96-2.89)	<.001
High risk vs low risk	20.4 (13.49-30.83)	<.001	4.81 (1.69-13.66)	.003	3.7 (2.68-5.10)	<.001
SES quantile, %^b^						
0-10						
Medium risk vs low risk	5.66 (4.62-6.93)	<.001	2.64 (1.60-4.36)	<.001	2.61 (2.22-3.07)	<.001
High risk vs low risk	8.24 (6.44-10.55)	<.001	3.93 (2.07-7.44)	<.001	3.52 (2.82-4.39)	<.001
10-30						
Medium risk vs low risk	8.92 (7.62-10.45)	<.001	4.99 (3.46-7.19)	<.001	2.59 (2.30-2.92)	<.001
High risk vs low risk	10.55 (8.67-12.84)	<.001	7.14 (4.50-11.34)	<.001	3.51 (2.98-4.13)	<.001
30-50						
Medium risk vs low risk	7.85 (6.73-9.15)	<.001	3.75 (2.56-5.50)	<.001	2.35 (2.08-2.64)	<.001
High risk vs low risk	10.05 (8.25-12.25)	<.001	3.94 (2.35-6.58)	<.001	3.27 (2.76-3.86)	<.001
50-70						
Medium risk vs low risk	7.1 (5.98-8.43)	<.001	3.34 (2.24-4.97)	<.001	3.07 (2.70-3.50)	<.001
High risk vs low risk	10.21 (8.27-12.61)	<.001	4.06 (2.44-6.78)	<.001	3.82 (3.18-4.59)	<.001
70-90						
Medium risk vs low risk	8.83 (7.38-10.58)	<.001	6.25 (4.14-9.43)	<.001	3.23 (2.8-3.73)	<.001
High risk vs low risk	10.68 (8.54-13.36)	<.001	10.33 (6.15-17.37)	<.001	4.37 (3.60-5.32)	<.001
90-100						
Medium risk vs low risk	7.72 (5.90-10.1)	<.001	12.32 (7.21-21.05)	<.001	3.35 (2.73-4.12)	<.001
High risk vs low risk	9.04 (6.50-12.58)	<.001	15.79 (7.68-32.46)	<.001	4.79 (3.65-6.29)	<.001

Abbreviations: aOR, adjusted odds ratio; CCI, Charlson Comorbidity Index; HFRS, Hospital Frailty Risk Score; RCRI, Revised Cardiac Risk Index; SES, socioeconomic status.

^a^ Adjusted for sex, age, individual components of the CCI, RCRI as a categorical variable, and surgical risk as categorical variable.

^b^ Lowest quantile indicates lowest SES.

## Discussion

This study found an association of frailty, as determined by the HFRS, with increased in-hospital, 30-day, and 1-year mortality using a large cohort of patients who underwent elective noncardiac surgery. Across all subgroups of sex, age group, surgical risk, and SES, a higher HFRS was associated with increased mortality. Although previous studies^2,7^ have found an association of age, sex, SES, and surgical complexity with surgical outcomes, we highlight that frailty as determined by HFRS is also an important consideration across these subgroups. Our study is limited by the reliance on *ICD-9* and *ICD-10* coding, which has inherent issues with accuracy and depth of coding to accurately define frailty. In addition, interactions between HFRS and other significant factors associated with mortality were not assessed. Implementation of an automated method available in medical records to classify patients’ frailty may allow clinicians and patients to have an informed discussion about the independent risks of increased frailty on postoperative outcomes and requires further investigation.
